# Desarrollo y patente de un sistema de aspiración intrapericárdico flexible

**DOI:** 10.47487/apcyccv.v3i3.231

**Published:** 2022-09-30

**Authors:** Necemio Arnaldo Aranda-Pretell

**Affiliations:** 1 Servicio de Cirugía Cardiovascular, Instituto Nacional Cardiovascular, EsSalud. Lima, Perú. Servicio de Cirugía Cardiovascular Instituto Nacional Cardiovascular, EsSalud Lima Perú

Señor editor

El desarrollo de la cirugía cardiaca y de los procedimientos cardiovasculares intervencionistas ha crecido sustancialmente en los últimos años. La cirugía cardiaca mínimamente invasiva, por ejemplo, ha revolucionado la filosofía de los cirujanos, obligándolos a reinventarse y a tratar de innovar constantemente, no solo en el ámbito de la técnica *per se*, sino también en el desarrollo de nuevas tecnologías e instrumentos quirúrgicos que permitan realizar dichas técnicas ^1^^,^^2^.

**Figura 1:**
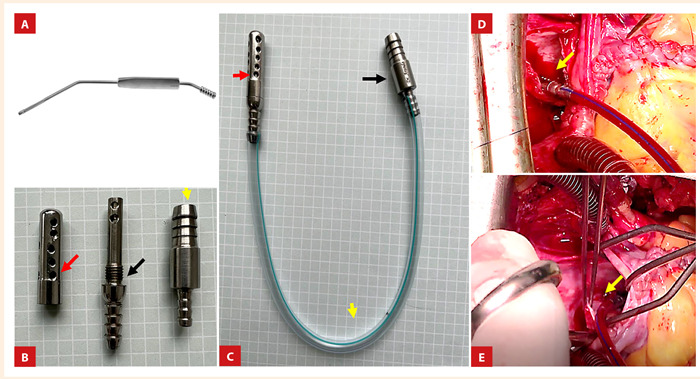
A. Aspirador intra-pericárdico de acero quirúrgico disponible en el mercado. B. Nuestro sistema de aspiración y su camiseta (flecha roja y negra), dispositivo conector (flecha amarilla), C. Nuestro sistema de aspiración: dispositivo terminal de aspiración (flecha roja) y dispositivo conector hacia la tubuladura (flecha negra) unidos por una sonda de poliestireno (flecha amarilla). D, E. Uso del sistema de aspiración en una cirugía de reducción de aurícula izquierda (flecha amarilla), se puede notar su practicidad y ductilidad.

Como es ampliamente conocido, para realizar la cirugía a «corazón abierto» se requiere de un circuito de circulación extracorpórea (CEC), el cual, dentro de todo su equipamiento, tiene un sistema de aspiración que permite recolectar la sangre desde la cavidad pericárdica y reingresarla a la circulación luego de pasar por un sistema de filtros. Este sistema requiere de aspiradores que, en la mayoría de los casos, son reusables y de acero quirúrgico, por lo tanto, rígidos (Figura 1A). La rigidez de estos aspiradores genera que sea difícil de usarlos en cavidades pequeñas (por ejemplo, aspirar dentro del ventrículo izquierdo a través de una atriotomía izquierda) o en la cirugía miniinvasiva.

Este constante problema nos llevó a inventar y patentar un sistema de aspiración «flexible» que pueda ser usado con facilidad a través de orificios pequeños y en la cirugía miniinvasiva. Además de práctico, que fuera reusable, es decir, de acero quirúrgico.

En la Figura 1 se observa nuestro innovador sistema de aspiración; este consta de dos dispositivos de acero quirúrgico (Figura 1C), uno de ellos se conecta a los tubos de aspiración intrapericárdica del sistema de CEC (Figura 1C, flecha negra), y el otro ingresa a la cavidad pericárdica o dentro de las cavidades cardíacas (Figura 1C, flecha roja). A su vez, el dispositivo de aspiración tiene una camiseta desmontable que, además de proporcionar una aspiración continua y homogénea de la sangre, permite su adecuado lavado y esterilización (Figura 1B, flecha roja y negra). Los dos dispositivos se unen entre sí con una sonda flexible de polietileno (sonda Nélaton o sonda de aspiración, número 14), la cual es desechable (Figura 1C, flecha amarilla). Este mecanismo nos ofrece un eficaz y cómodo sistema de aspiración intracardiaca, ya que por su ductilidad puede ingresar fácilmente en las cavidades del corazón, además de ser muy útil en cirugías en espacios reducidos (mínimamente invasivas) (Figura 1D, E).

Las características de este dispositivo aspirador flexible de transformar la aspiración intermitente con el aspirador rígido en una aspiración continua y permanente hacen que la cirugía siga de continuo sin interferencias y, en consecuencia, disminuye el tiempo operatorio
